# Roles of plant‐based ingredients and phytonutrients in canine nutrition and health

**DOI:** 10.1111/jpn.13626

**Published:** 2021-09-08

**Authors:** Jirayu Tanprasertsuk, Devon E. Tate, Justin Shmalberg

**Affiliations:** ^1^ NomNomNow Inc Nashville TN USA; ^2^ Department of Comparative, Diagnostic and Population Medicine College of Veterinary Medicine University of Florida Gainesville FL USA

**Keywords:** carotenoids, dogs, phytochemicals, phytonutrients, polyphenols

## Abstract

Dogs possess the ability to obtain essential nutrients, established by the Association of American Feed Control Officials (AAFCO), from both animal‐ and plant‐based ingredients. There has been a recent increase in the popularity of diets that limit or completely exclude certain plant‐based ingredients. Examples of these diets include ‘ancestral’ or ‘evolutionary’ diets, raw meat‐based diets and grain‐free diets. As compared to animal sources, plant‐derived ingredients (including vegetables, fruits, grains, legumes, nuts and seeds) provide many non‐essential phytonutrients with some data suggesting they confer health benefits. This review aims to assess the strength of current evidence on the relationship between the consumption of plant‐based foods and phytonutrients (such as plant‐derived carotenoids, polyphenols and phytosterols) and biomarkers of health and diseases (such as body weight/condition, gastrointestinal health, immune health, cardiovascular health, visual function and cognitive function) from clinical trials and epidemiological studies. This review highlights the potential nutritional and health benefits of including plant‐based ingredients as a part of balanced canine diets. We also highlight current research gaps in existing studies and provide future research directions to inform the impact of incorporating plant‐based ingredients in commercial or home‐prepared diets.

## 1 INTRODUCTION

Dogs have the ability to consume and utilize energy from both animal‐based products (meat, organ meat, eggs and dairy products) and a variety of edible plant‐based foods (fruits, vegetables, legumes, grains, nuts and seeds). The National Research Council (NRC) of the U.S. National Academy of Sciences provides a summary of the evidence and basis of essential nutrient recommendations for dogs; these are largely incorporated into the regulatory guidelines of the Association of American Feed Control Officials (AAFCO), which establishes nutrient profiles for different life stages, including adult dogs for maintenance, growth and/or reproduction (AAFCO, 2019; NRC, 2006). All commercial dog foods sold in interstate commerce in the United States are typically required to meet the standards established by AAFCO. However, unlike the dietary guidelines for humans, such as the Dietary Guidelines for Americans (U.S. Department of Health & Human Services & U.S. Department of Agriculture [USDA], 2015), no official guidelines have been established to recommend which dietary sources dogs should obtain the required nutrients from.

Dog owners encounter a litany of dietary options, and dogs display wide dietary flexibility (Bradshaw, 2006). On one hand, plant‐based diets are increasing in popularity, especially among vegan dog owners (Dodd et al., 2019), and for dogs with specific health conditions such as food allergies. On the contrary, recent diet trends limit or exclude plant‐based ingredients, suggesting that some owners prefer diets with meat, organ meat and bones as the main ingredients (Beloshapka et al., 2013; Kim et al., 2017; Sandri et al., 2017). Examples of such popular diets include the grain‐free diet (Mansilla et al., 2019), the ancestral diet (Brown, 2009), the bones and raw food diet (BARF, also known as the biologically appropriate raw food diet) (Billinghurst, 1993), based on a perception that plant‐based foods are ‘fillers’ or indigestible inclusions with inferior nutrient composition (Beynen, 2014). Meanwhile, these heavily meat‐based diets have raised health concerns in the veterinary community because some of these diets, including those which are home‐prepared and some commercial formulations (Freeman & Michel, 2001), were nutritionally incomplete (Dillitzer et al., 2011; Freeman et al., 2013; Freeman & Michel, 2001). Many of these diets did not meet the minimum requirements for calcium, zinc, copper, potassium, magnesium, manganese, and vitamins A and D (Dillitzer et al., 2011; Freeman & Michel, 2001).

Given the lack of current recommendations on dietary sources of nutrients, and the recent diet trends that limit the consumption of plant‐based ingredients, it is timely to summarize existing evidence for the nutritional and health benefits of including plant‐based foods in the canine diet. Further, given that diseased dogs may have different nutritional requirements from generally healthy dogs, the therapeutic roles of diet in the diseased population are not a primary focus of this review, but is discussed when evidence in the healthy population is limited. The scope of this review includes studies where compounds and nutrients, such as vitamins C and E and essential fatty acids, were used in a natural plant‐based food form or in isolation as extracts or supplements, but note that differences in bioavailability between different ingredient sources exist. Given their merit, studies using combined nutrients that included both plant‐ and animal‐based ingredients are also mentioned, but it is noted that the isolated impact of plant‐based treatment cannot be determined in these studies. We also excluded from this review foods and compounds that are toxic to dogs at dietary doses (Kovalkovičová et al., 2009), as well as plants, herbs and phytonutrients that are not recognized as Generally Recognized as Safe (GRAS) ingredients by AAFCO (Thompson, 2008). Additionally, while we recognize the expansive health impacts of plant‐based dietary fibres, they have been extensively reviewed elsewhere (Wernimont et al., 2020) and are thus not included within the scope of this review.

## 2 PHYTONUTRIENTS AND SELECTED DIETARY SOURCES

Phytonutrients are a group of compounds that are naturally present in dietary plants. They are not essential in the canine diet; therefore, no dietary requirements have been established, and absence of these nutrients will not induce a deficiency state (Rahal et al., 2014). Intake of phytonutrients may confer health benefits as discussed in the following sections. To our knowledge, the average intake of phytonutrients in the general dog population has not been estimated, but it is likely dependent on the owner's choice of diet, treats and dietary supplements. All ingredients used in conforming commercial dog foods sold in interstate commerce in the United States typically follow AAFCO guidelines (Thompson, 2008). The list of regulated ingredients and their definitions are annually updated and include, but are not limited to, products of fruits, vegetables, grains, legumes, nuts and seeds (AAFCO, 2019), all of which are sources of phytonutrients in commercial dog foods. For example, an analysis of commercial dog foods containing soya beans or soya bean fractions showed that in 11 out of the 12 foods, soy phytonutrients were detected (soy isoflavones; daidzein ranging from 24 to 615 µg/g and genistein ranging from 4 to 238 µg/g of dry matter), while none were detected in foods without soya bean listed as an ingredient (Cerundolo et al., 2004).

Some examples of common phytonutrients are dietary fibres, carotenoids, polyphenols and phytosterols. Selected dietary sources of various phytonutrients (excluding dietary fibres) are provided in Table 1.

**Figure 1:**
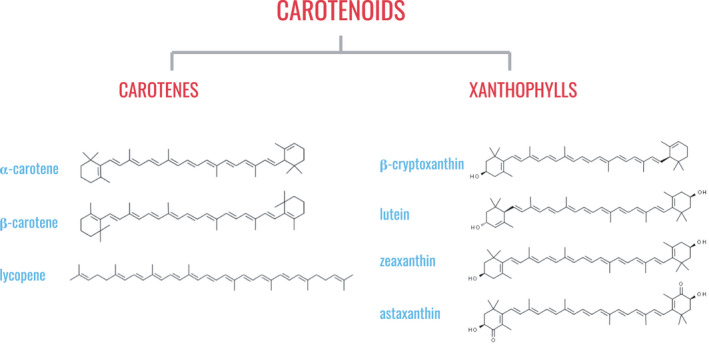
Classification of carotenoids based on chemical structure and examples of common dietary carotenoids. Most carotenoids in plants are composed of 40 branched carbon units and conjugated double bonds. Carotenoids can be classified as carotenes (containing no oxygen atoms) and xanthophylls (containing one or more oxygen atoms). [Colour figure can be viewed at wileyonlinelibrary.com]

**Figure 2:**
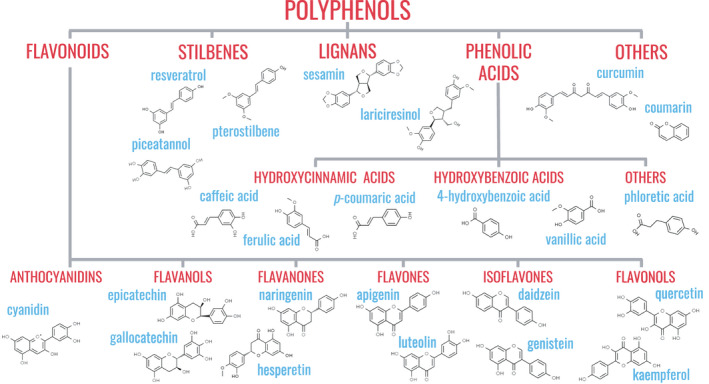
Classification of polyphenols based on chemical structure and examples of common dietary polyphenols. Polyphenols are compounds characterized by two or more aromatic rings, each of which contains one or more phenol groups, linked together. They are broadly classified based on their chemical structures into four or more classes. Flavonoids contain a backbone of two benzene rings linked by a chain containing three carbon atoms, which joins to form a heterocycle in which oxygen is the non‐carbon atom. Flavonoids are divided further into six subclasses based on their structures which are anthocyanidins, flavanols, flavanones, flavones, isoflavones and flavonols. Stilbenes are characterized by their distinctive C6‐C2‐C6 unit, with the C6 being a benzene ring and the C2 being an ethylene group. Lignans are a group of polyphenols with their basic chemical structure consisting of two phenylpropane units linked by a C‐C bond between the β position of the propane group. Phenolic acids comprise a benzene ring with at least one hydroxyl group attached to it, and can further be divided into subclasses including hydroxycinnamic acids and hydroxybenzoic acids. [Colour figure can be viewed at wileyonlinelibrary.com]

**Figure 3:**
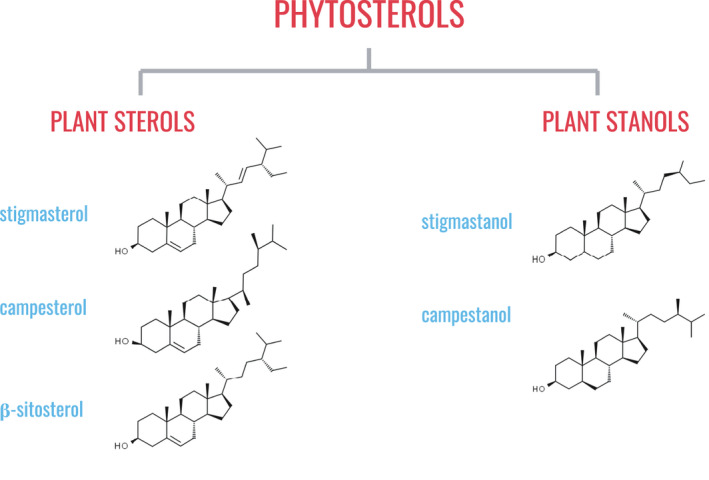
Classification of phytosterols based on chemical structure and examples of common dietary phytosterols. Like cholesterol and other steroids, phytosterols are characterized by their cyclopentanoperhydrophenanthrene carbon backbone, which consists of three six‐carbon rings and one five‐carbon ring. Plant sterols have a double bond at the C5 position while plants stanols do not. [Colour figure can be viewed at wileyonlinelibrary.com]

● *Dietary fibres*. Dietary fibres are a group of carbohydrates that cannot be directly used for energy by mammals due to a lack of digestive enzymes to break down structural linkages, such as the β‐1,4‐glycosidic bonds found in cellulose. While fibres are not within the scope of this review, their impacts on the health status of dogs are widely known (Wernimont et al., 2020), in particular their influence on body condition, gastrointestinal (GI) health, and immune parameters are broadly accepted.
● *Carotenoids*. Carotenoids are fat‐soluble pigments found in yellow, orange and red fruits and vegetables, as well as green leafy vegetables. More than 600 different carotenoids have been identified in nature, but the six most common carotenoids in common fruits and vegetables are α‐carotene, β‐carotene, cryptoxanthin, lycopene, lutein and zeaxanthin (Figure 1) (Ford, 2000; Olson & Krinsky, 1995). They are also available in a dietary supplement form for dogs, including those less commonly found in food sources such as astaxanthin. Additionally, β‐carotene, lycopene, astaxanthin and canthaxanthin may be used as colour additives in commercial dog foods (AAFCO, 2019). α‐Carotene, β‐carotene and cryptoxanthin are provitamin A carotenoids, meaning that they can be cleaved by the enzyme β‐carotene 15,15′‐oxygenase and yield one or two vitamin A molecules (Green & Fascetti, 2016).
● *Polyphenols*. Polyphenols are broadly classified based on their chemical structures into four or more classes (Figure 2) (Pandey & Rizvi, 2009):
  ○ *Phenolic acids*. Common phenolic acids include hydroxycinnamic acids (e.g. caffeic acid, ferulic acid and sinapic acid), hydroxybenzoic acids, hydroxyphenylacetic acids and hydroxyphenylpropanoic acids (Zamora‐Ros et al., 2013).
  ○ *Flavonoids*. Most flavonoids naturally exist as glycosides (sugar‐linked) or other conjugates, and can be polymerized by plants or food processing into large molecules collectively called tannins. Flavonoids are divided further into six subclasses based on their structures (Beecher, 2003; Panche et al., 2016):
    ■ *Anthocyanidins*. Examples include cyanidin, which is responsible for the distinct colours of berry fruits such as blueberries and red sweet cherries.
    ■ *Flavanols*. Epigallocatechin and catechin are some common flavanols which are found in green tea (*Camellia sinensis*) and may confer the benefits of tea consumption in humans (Ferruzzi et al., 2019). Flavanols are also found widely in common fruits and vegetables, as well as wine and cocoa.
    ■ *Flavanones*. Common dietary flavanones are naringenin, hesperetin and eriodictyol, which are commonly found in citrus fruits.
    ■ *Flavones*. Various tea and dry herbs such as oregano, parsley and rosemary are common sources of flavones (Hostetler et al., 2017). Some common flavones include apigenin, luteolin, acacetin and chrysoeriol.
    ■ *Isoflavones*. Soya bean and soya bean products are significant sources of isoflavones daidzein and genistein. Small amounts of isoflavones are also found in other legumes.
    ■ *Flavonols*. Flavonols are the most ubiquitous flavonoids in various fruits and vegetables. Quercetin and kaempferol are the main flavonols widely investigated for their potential health benefits.
  ○ *Lignans*. Their basic chemical structure consists of two phenylpropane units linked by a C‐C bond between the β position of the propane group. Significant plant sources of lignans are flax and sesame seeds (Touré & Xueming, 2010). Minimal amounts are also found in fruits, vegetables and cereals. Lignans, with or without melatonin, are often prescribed as a treatment of atypical Cushing's disease in dogs (Fecteau et al., 2011), although there is little scientific support for the practice at this time.
  ○ *Stilbenes*. Stilbenes are characterized by their distinctive C6‐C2‐C6 unit, with the C6 being a benzene ring and the C2 being an ethylene group (thus also known as 1,2‐diphenylethylene). One of the most common stilbenes investigated for health benefits is resveratrol, which is found in red and black grapes, and their products. While grapes are generally toxic to dogs at dietary doses (Kovalkovičová et al., 2009), resveratrol has been shown to be safe at 200 mg/kg body weight daily (Mathew et al., 2018). Other stilbenes include piceatannol and which are found mostly in grapes and berry fruits.
● *Phytosterols*. Phytosterols include plant sterols and plant stanols and are structurally related to cholesterol (Figure 3). Common plant sterols in human diets are β‐sitosterol, campesterol and stigmasterol. Plant stanols are present in lesser amounts in terms of total dietary phytosterols, and include compounds such as stigmastanol and campestanol. Plant oils are significant sources of phytosterols but some amount can be found in fruits, vegetables, nuts, beans and seeds (Weihrauch & Gardner, 1978; Yang et al., 2019).

**Table 1:** Phytonutrients and selected plant‐based dietary sources that are safe for dogs (Normén et al., 2007; Rothwell et al., 2013; Touré & Xueming, 2010; USDA ARS NDL, 2015; Yang et al., 2019)

Phytonutrients	Selected dietary sources		Overview of health benefits for dogs
	Food	Amount	
Carotenoids (in mg/1000 kcal)			
α‐Carotene	Pumpkin	155	Body weight and body condition: A supplement containing carotenoids (β‐carotene, lutein and zeaxanthin) given to puppies tended to reduce fat mass and improved fat oxidation and lipid profiles (Wang et al., 2017). Insulin sensitivity and glycaemic control: Use of annatto (high carotenoid content) suppressed the postprandial rise in blood glucose level and increased plasma insulin level after an oral glucose load (Russell et al., 2005). Cardiovascular outcomes: β‐Carotene, lutein and α‐tocopherol given together increased plasma antioxidant concentrations and decreased oxidative damage post‐exercise (Baskin et al., 2000). Immune health: In Beagle dogs fed varying levels β‐carotene, plasma antibody immunoglobulin G concentrations were found to increase dose‐dependently (Chew, Park, Weng, et al., 2000; Chew, Park, Wong, et al., 2000). Dogs provided β‐carotene showed higher CD4+ T‐cell levels and displayed increased delayed‐type hypersensitivity (DTH) response to both specific and non‐specific immune response triggers (Chew, Park, Weng, et al., 2000; Chew, Park, Wong, et al., 2000). In older dogs supplemented with β‐carotene, immunological variables were altered by increasing levels of CD4+ T cells, improving T‐cell proliferation, and heightening DTH responses (Massimino et al., 2003). Yellow‐orange vegetables and green leafy vegetables were significantly associated with a decrease in risk of developing transitional cell carcinoma of the urinary bladder in Scottish Terriers (Raghavan et al., 2005). Lutein provided to female Beagle dogs displayed immune‐modulating effects, by enhancing DTH to immune response triggers, increasing immunoglobulin G production and increasing the population of several lymphocyte subsets (Kim et al., 2000). A diet consisting of spinach flakes, tomato pomace, grape pomace, carrots, citrus pulp and other antioxidative nutrients in combination with behavioural enrichment, served to increase neutrophil phagocytosis and B‐cell populations in aged Beagles (Hall et al., 2006). Bone and joint health: Treatment of canine osteosarcoma cells with lycopene reduced cell proliferation and induced apoptosis of different cell lines to varying degrees (Wakshlag & Balkman, 2010). Renal health: Supplementation with vitamin E and carotenoids reduced proteinuria, glomerulosclerosis and interstitial fibrosis in Beagle dogs (Brown, 2008). A diet consisting of beet pulp, citrus pulp, carrot granules, dried spinach, tomato pomace along with other antioxidative nutrients significantly decreased levels of SDMA and creatinine, and improved canine renal function (Hall, MacLeay, et al., 2016; Hall, Yerramilli, et al., 2016). A traditional renal protective food diet was provided to aged dogs and supplemented with functional foods, including beet pulp, citrus pulp, carrot granules, dried spinach, tomato pomace and other antioxidative nutrients; serum SDMA decreased in those supplemented with functional foods (Hall, MacLeay, et al., 2016; Hall, Yerramilli, et al., 2016). Visual health: A daily dose of lutein, zeaxanthin, β‐carotene, astaxanthin, vitamin C and vitamin E significantly improved retinal responses in Beagle dogs with healthy eyes (Wang et al., 2016). Cognitive Health: A diet containing spinach flakes, tomato pomace, grape pomace, carrot granules, citrus pulp and other antioxidative ingredients was used in several cognitive trials. It showed that dogs receiving the fortified diet learned the landmark discrimination learning task more rapidly (Milgram, Head, et al., 2002; Milgram, Zicker, et al., 2002), and performed better on the oddity discrimination learning task at higher levels (Milgram, Head, et al., 2002; Milgram, Zicker, et al., 2002). Additionally, those receiving the fortified diet in combination with a behavioural intervention performed better on size discrimination reversal‐learning task (Milgram et al., 2004), and on several learning tasks in a two‐year follow‐up (Milgram et al., 2005).
	Carrots	85	
	Butternut squash	28	
β‐Carotene	Spinach	245	
	Carrots	202	
	Sweet potatoes	124	
	Pumpkin	119	
	Butternut squash	114	
	Kale	82	
	Cantaloupe	59	
	Red sweet peppers	52	
β‐Cryptoxanthin	Butternut squash	78	
	Red sweet peppers	16	
	Papayas	14	
	Tangerines	8	
Lycopene	Watermelons	151	
	Tomatoes	143	
	Papayas	43	
Lutein + Zeaxanthin	Spinach	530	
	Kale	179	
	Collard greens	135	
	Green peas	31	
	Asparagus	36	
	Green beans	21	
	Sweet yellow corn	9	
Polyphenols (in mg/1000 kcal)			
*Phenolic acids* a			Body weight and body condition: Dogs fed diets with 25% higher than required calories, supplemented with soy isoflavones, gained significantly less weight than dogs not receiving soy isoflavones (Pan, 2006). Overweight dogs consuming soy isoflavones were more likely to achieve weight loss goals than dogs on diets without soy isoflavones (Pan et al., 2008). Green tea polyphenols were shown to attenuate the impacts of a high‐fat diet on weight gain and inflammation in healthy male Beagles (Rahman et al., 2020). Consumption of green tea polyphenols was found to have a positive impact on weight status, inflammation and gut microbiota populations in dogs (Li et al., 2020). Insulin sensitivity and glycaemic control The polyphenol content of rosemary and basil reduced fasting glucose levels in Rottweiler dogs (Abdelrahman et al., 2020). Green tea polyphenols improved the insulin sensitivity index by 60% in obese dogs (Serisier et al., 2008). GI health and the gut microbiota Grape proanthocyanidins were demonstrated to alter the abundances of select faecal microbiota populations and SCFAs in healthy adult dogs (Scarsella et al., 2020). Polyphenol supplementation from pomegranate peel extract increased faecal concentrations of total SCFAs and fermentative metabolites, and improved antioxidant status in healthy dogs (Jose et al., 2017). Supplementation with green tea polyphenols altered the structure of the gut microbiota in adult male dogs (Li et al., 2020). Cardiovascular outcomes: Blueberries were shown to attenuate post‐exercise oxidative damage and elevate antioxidant status in healthy sled dogs (Dunlap et al., 2006). Quercetin, a flavonol, given at 50 mg/kg was found to be cardioprotective in dogs given an experimental myocardial infarction (Kolchin et al., 1991). Purple grape juice, high in flavonoids quercetin, kaempferol and myricetin, given to dogs with coronary stenosis, was shown to have antithrombotic effects (Demrow et al., 1995; Osman et al., 1998). Grape seed and skin extracts given together inhibited platelet aggregation in healthy dogs (Shanmuganayagam et al., 2002). Resveratrol, and other antioxidants, from a formulation containing blueberry, strawberry, blackberry and grape seed extracts significantly reduced exercise‐induced oxidative stress (Sechi et al., 2017). Bone and joint health: Curcumin provided to dogs with osteoarthritis (OA) was found to provide supplemental anti‐inflammatory actions (Colitti et al., 2012). Supplementation of turmeric extract (6.6 mg/kg body weight of curcumin) led to a downregulation of inflammatory genes in circulating white blood cells in dogs with a history of OA (Sgorlon et al., 2016). Use of P54FP, an extract of turmeric containing curcumoids, was used in the treatment of dogs with OA, and study investigators, but not owners, noted improvement (Innes et al., 2003). Treatment of canine osteosarcoma cells with the flavonoid baicalein reduced cell proliferation and induced apoptosis of different cell lines to varying degrees (Helmerick et al., 2014). Avocado/soya bean unsaponifiables (ASU) provided to dogs with experimental OA were found to improve structural changes associated with the early stages of OA (Boileau et al., 2009). ASU supplementation increased cytokine TGF‐β_1_ and TGF‐β_2_ levels in the synovial fluid of healthy dogs, which are linked to cartilage synthesis (Altinel et al., 2007). The flavonoid myricetin was shown to induce apoptosis in canine osteosarcoma cells (Park et al., 2018). Skin and coat health: An *in vitro* study using a nutrient combination that included curcumin demonstrated promise in helping to maintain the canine skin barrier (Fray et al., 2004). Healthy dogs fed high‐ or low‐isoflavone soy‐based diets, had no detectable differences in skin and coat health, indicating soy as a safe ingredient for dogs with skin and hair issues (previously thought to have negative impact) (Cerundolo et al., 2009). Both healthy dogs supplemented with flax or sunflower seeds showed improvements in their skin and coat condition scores after 1 month (Rees et al., 2001). Black currant seed oil given to dogs with atopic dermatitis showed nonsignificant clinical improvements (Noli et al., 2007). An *in vitro* study showed that a nutraceutical mixture containing phenolic compounds downregulated the expression of inflammatory genes in inflamed canine keratinocytes and monocytes (Massimini et al., 2021). Cognitive Health: Healthy aged Beagles receiving mixed grape and blueberry extract showed improvements in their working memory (Fragua et al., 2017).
Hydroxybenzoic acids	Cranberries	1152	
	Strawberries	188	
	Raspberries	96	
	Blueberries	18	
Hydroxycinnamic acids	Plums	1935	
	Blueberries	1526	
	Cherries	1397	
	Sunflower seeds	793	
	Peaches	641	
	Apples	346	
	Potatoes	326	
	Oat flour	30	
*Flavonoids* b			
Anthocyanidins	Blueberries	2860	
	Cranberries	1478	
	Strawberries	844	
	Cherries	508	
Flavanols	Cherries	159	
	Strawberries	156	
	Cranberries	152	
	Apples	135	
	Bananas	67	
Flavanones	Navel oranges	592	
	Tangerines	340	
Flavones	Parsley	6028	
	Celery	250	
Isoflavones	Soy meal	642	
	Tofu	492	
	Soy flour	410	
	Tempeh	313	
	Soya beans	287	
Flavonols	Kale	2657	
	Asparagus	1050	
	Cranberries	522	
	Spinach	478	
	Broccoli	324	
	Blueberries	193	
	Kidney beans	56	
Lignans^c^	Sesame seed meal	1369	
	Flaxseed meal	533	
	Pumpkin	4	
	Yellow sweet peppers	3	
Stilbenes^d^	Strawberries	11	
	Lentils	0.8	
	Peanuts	0.1	
Phytosterols^e^ (in mg/1000 kcal)	Wheat germ oil	1094	Cardiovascular outcomes and insulin sensitivity: Phytosterols in beans are suspected to be responsible for the cholesterol‐lowering effects, with overweight dogs receiving dry black or navy bean powder decreasing serum total cholesterol. Navy bean consumption also led to a decrease of serum triglyceride levels (Forster et al., 2012). Navy beans lowered serum cholesterol in healthy adult dogs (Forster et al., 2015). Dogs gaining weight on a corn oil diet had a small increase in mean arterial pressure and no change in insulin sensitivity, while those on a lard diet experienced a large increase in mean arterial pressure and some insulin resistance (Truett et al., 1998).
	Corn oil	1087	
	Oat oil	604	
	Flaxseed oil	533	
	Almonds	359	
	Cashew nuts	273	
	Peanuts	205	
	Black beans	149	

^a^ Hydroxybenzoic acids include 2,4‐dihydroxybenzoic acid, 3‐hydroxybenzoic acid, 4‐hydroxybenzoic acid, benzoic acid, ellagic acid, galloylquinic acid, vanillic acid and their glucosides. Hydroxycinnamic acids are calculated from avenanthramide, 3‐caffeoylquinic acid, 3‐feruloylquinic acid, 3‐p‐coumaroylquinic acid, 4‐caffeoylquinic acid, 4‐p‐coumaroylquinic acid, 5‐caffeoylquinic acid, 5‐p‐coumaroylquinic acid, caffeic acid, ferulic acid, p‐coumaric acid, sinapic acid.

^b^ Anthocyanidins are calculated from cyanidin, petunidin, delphinidin, malvidin, pelargonidin, peonidin. Flavanols are calculated from (+)‐catechin, (−)‐epigallocatechin, (−)‐epicatechin, (−)‐epicatechin 3‐gallate, (−)‐epigallocatechin 3‐gallate, (+)‐gallocatechin. Flavanones are calculated from hesperetin and naringenin. Flavones are calculated from apigenin and luteolin. Flavonols are calculated from isorhamnetin, kaempferol, myricetin, quercetin.

^c^ Lignans are calculated from isohydroxymatairesinol, sesamin, sesaminol, sesamolin, lariciresinol, matairsinol, pinoresinol, secoisolariciresinol.

^d^ Stilbenes are calculated from resveratrol and resveratrol 3‐O‐glucoside.

^e^ Phytosterols are the sum of campesterol, campestanol, stigmasterol, sitosterol, sitostanol, brassicasterol, 5‐avenasterol.

The bioavailability of phytonutrients has been limitedly investigated in dogs, although there is some evidence of absorption, distribution and metabolism. Carotenoids, lutein, β‐carotene and astaxanthin were detected dose‐dependently in the circulation following oral administration in Beagles (Chew, Park, Weng, et al., 2000; Chew, Park, Wong, et al., 2000; Park et al., 2010; Shanmugam et al., 2011). Carotenemia, a harmless clinical condition causing skin to turn yellow as a result of prolonged consumption of β‐carotene, has also been observed in dogs (Duhig, 1931). Additionally, adding 150 g/d of carrots (a significant source of β‐carotene) to a vitamin A‐deprived canine diet consisting of lamb meat and boiled rice was shown to prevent death from vitamin A deficiency (Turner, 1934). Collectively, these results suggest that dogs can absorb, store and metabolize carotenoids at dietary doses (Green & Fascetti, 2016).

Similarly, quercetin, a flavonol found in green leafy vegetables and often supplemented in commercial dog foods, was detected in the plasma of Beagle dogs within an hour post oral administration of quercetin or its glycoside forms at 30 μmol/kg body weight (10 mg/kg body weight) (Reinboth et al., 2010). Resveratrol and its derivatives were detected in the plasma of healthy Beagles 8 hours after its oral administration at various pharmacological doses (Muzzio et al., 2012). Epigallocatechin gallate (EGCG), a phenolic compound derivative found in green tea, was also detected in various epithelial tissues of Beagles after daily consumption of 250 mg/kg body weight for 28 days (Swezey et al., 2003), but pharmacological doses were shown to be toxic in dogs after chronic consumption in a study lasting nine months (Kapetanovic et al., 2009). Evidence in rodent and human studies suggest that some intact polyphenols are absorbed directly in the small intestine, but most travel further to the large intestine where they are modified by the intestinal microbiota and later absorbed (Clavel et al., 2006; Kawabata et al., 2019; Ozdal et al., 2016). The metabolism of polyphenols by microbes in the canine digestive tract remains largely uninvestigated, but evidence of their bioavailability is emerging.

## 3 ROLES OF PLANT‐BASED FOODS IN MEETING CANINE NUTRIENT REQUIREMENTS

As shown in Table 2, essential nutrient minima for maintenance of adult dogs per 1000 kcal for commercial diets by the 2019 AAFCO Official Publication have been established, which contains some modifications from the scientific recommendations of the NRC (AAFCO, 2019). It is possible to meet certain requirements from feeding either animal‐ or plant‐based foods, or a combination of both, although the digestibility of sources may be different. Examples are as follows:

● *Vitamin A*. A daily recommended allowance of vitamin A has been established by the NRC (1263 IU/1000 kcal metabolizable energy (ME) diet) and a minimum by AAFCO (1250 IU/1000 kcal ME diet) for adult dogs. This requirement in dogs can be met from the consumption of either preformed vitamin A found in meat and organ meat, or as provitamin A carotenoids present in fruits and vegetables, which can be endogenously converted to vitamin A. For dogs, a retinol equivalency has not been clearly defined; however, it is apparent they can utilize provitamin A carotenoids, notably β‐carotene, as their exclusive vitamin A source (Green & Fascetti, 2016; McDowell, 2000). It is expected that, as is true in humans, the food matrix and genetics may impact conversion efficiency (McDowell, 2000).
● *Amino acids*. Both plant‐based ingredients and meat are sources of essential AAs, although in general methionine content is relatively low in vegetables and legumes, and lysine content is relatively low in grains and nuts (USDA Agriculture Research Service [ARS] Nutrient Data Laboratory [NDL], 2015). As shown with theoretical examples in Table 2, it is possible to meet the recommended intake for all essential AAs from consuming turkey meat and skin alone, or a combination of 90% kcal from turkey meat and skin and 10% kcal from selected plant foods in hypothetical unbalanced diets (spinach, mushrooms, peas, blueberries and brown rice as examples). Nonetheless, amino acid digestibility must also be considered. Pulse‐based diets containing higher plant protein and lower animal protein were shown to have lower amino acid digestibility in a recent study (Quilliam et al., 2021). Plasma sulphur‐containing amino acid concentrations (methionine, cysteine and taurine), however, were not compromised after dogs were on pulse‐based diets for 7 days.
● *Fatty acids*. Substituting 10% kcal of turkey meat and skin with plant foods does not place the amount of total fats and linoleic acid lower than the recommended minimums but does increase the amounts of other essential nutrients and provide additional non‐essential nutrients as discussed below.

**Table 2:** The AAFCO minimum nutrient requirements per 1000 kcal metabolizable energy (ME) diet for adult dogs and nutrient profiles in foods per 1000 kcal serving (% of AAFCO values)

Nutrients	AAFCO minimum per 1000 kcal ME diet	1000 kcal diet 100% cooked turkey meat and skin		1000 kcal diet with 90% kcal ME from cooked turkey meat and skin									
				10% kcal ME from spinach		10% kcal ME from mushrooms		10% kcal ME from peas		10% kcal ME from blueberries		10% kcal ME from brown rice	
		Absolute Amount	% AAFCO minimum	Absolute Amount	% AAFCO minimum	Absolute Amount	% AAFCO minimum	Absolute Amount	% AAFCO minimum	Absolute Amount	% AAFCO minimum	Absolute Amount	% AAFCO minimum
**Crude protein (g)**	45.0	132.4	294%	131.6	292%	133.2	296%	125.5	279%	120.4	268%	121.4	270%
Arginine (g)	1.28	7.58	592%	7.52	588%	7.17	560%	7.32	572%	6.88	538%	6.98	545%
Histidine (g)	0.48	3.45	719%	3.38	704%	3.36	700%	3.23	673%	3.12	650%	3.16	658%
Isoleucine (g)	0.95	3.67	386%	3.94	415%	3.64	383%	3.53	372%	3.34	352%	3.39	357%
Leucine (g)	1.70	8.87	521%	8.95	526%	8.53	502%	8.36	492%	8.06	474%	8.16	480%
Lysine (g)	1.58	10.50	665%	10.21	646%	9.94	629%	9.83	622%	9.48	600%	9.53	603%
Methionine (g)	0.83	3.33	401%	3.23	389%	3.14	378%	3.09	372%	3.02	364%	3.04	366%
Methionine + Cystine (g)	1.63	4.55	279%	4.48	275%	4.29	263%	4.23	260%	4.13	253%	4.17	256%
Phenylalanine (g)	1.13	4.17	369%	4.31	381%	4.14	366%	3.99	353%	3.80	336%	3.86	342%
Phenylalanine + Tyrosine (g)	1.85	7.94	429%	8.17	442%	7.73	418%	7.51	406%	7.20	389%	7.33	396%
Threonine (g)	1.20	4.62	385%	4.69	391%	4.65	388%	4.40	367%	4.19	349%	4.24	353%
Tryptophan (g)	0.40	1.34	335%	1.37	343%	1.36	340%	1.25	313%	1.21	303%	1.23	308%
Valine (g)	1.23	4.17	339%	4.45	362%	4.81	391%	4.03	328%	3.81	310%	3.88	315%
**Total fats (g)**	13.8	48.3	350%	45.2	328%	45.0	326%	43.7	317%	44.0	319%	44.3	321%
Linoleic acid (g)	2.8	12.1	432%	11.0	393%	11.6	414%	11.0	393%	11.1	396%	11.2	400%
**Minerals**													
Calcium (mg)	1250	83	7%	**505**	**40%**	88	7%	106	8%	85	7%	77	6%
Phosphorus (mg)	1000	1044	104%	1152	115%	1330	133%	1078	108%	960	96%	1023	102%
Potassium (mg)	1500	1107	74%	**3422**	**228%**	**2442**	**163%**	1319	88%	1131	75%	1066	71%
Sodium (mg)	200	510	255%	802	401%	481	241%	462	231%	460	230%	462	231%
Chloride (mg)^a^	300	N/A	N/A	N/A	N/A	N/A	N/A	N/A	N/A	N/A	N/A	N/A	N/A
Magnesium (mg)	150	131	87%	**461**	**307%**	159	106%	164	109%	128	85%	150	100%
Iron (mg)	10.0	7.0	70%	**18.1**	**181%**	8.6	86%	8.2	82%	6.8	68%	6.8	68%
Copper (mg)	1.83	0.61	33%	1.11	61%	**1.99**	**109%**	0.75	41%	0.65	36%	0.63	34%
Manganese (mg)	1.25	0.08	6%	**3.97**	**318%**	**0.29**	**23%**	**0.70**	**56%**	**0.66**	**53%**	**0.87**	**70%**
Zinc (mg)	20	16	80%	17	85%	17	85%	16	80%	15	75%	15	75%
Iodine (μg)^a^	250	N/A	N/A	N/A	N/A	N/A	N/A	N/A	N/A	N/A	N/A	N/A	N/A
Selenium (μg)	80	147	184%	136	170%	174	217%	134	168%	132	165%	137	171%
**Vitamins**													
Vitamin A (IU)^b^	1250	228	18%	**21804**	**744%**	205	16%	**699**	**56%**	255	20%	205	16%
Vitamin D (IU)	125	73	58%	66	53%	97	78%	66	53%	66	53%	66	53%
Vitamin E (IU)^b^	12.5	0.6	5%	**13.7**	**110%**	0.6	5%	0.8	6%	**2.0**	**16%**	0.7	6%
Thiamin (mg)	0.56	0.28	50%	**0.59**	**105%**	**0.62**	**111%**	**0.56**	**100%**	0.31	55%	0.39	70%
Riboflavin (mg)	1.3	1.8	138%	2.4	185%	3.4	262%	1.8	138%	1.7	131%	1.7	131%
Niacin (mg)	3.4	34.5	1015%	34.2	1006%	47.4	1394%	33.4	982%	31.8	935%	33.1	974%
Pantothenic acid (mg)	3.0	4.9	163%	4.7	157%	**11.2**	**373%**	4.6	153%	4.6	153%	4.7	157%
Pyridoxine (mg)	0.38	2.09	550%	2.73	718%	2.35	618%	2.14	563%	1.97	518%	1.98	521%
Folate (μg)	54	44	81%	**883**	**1635%**	**117**	**217%**	**114**	**211%**	50	93%	47	87%
Choline (mg)	340	452	133%	491	144%	486	143%	443	130%	418	123%	415	122%
Cobalamin (μg)	7.0	8.1	116%	7.3	104%	7.4	106%	7.3	104%	7.3	104%	7.3	104%

Note

The recommended intakes for all essential amino acids and fats are met from consuming turkey meat and skin alone, or a combination of 90% kcal from turkey meat and skin and 10% kcal from selected plant foods in hypothetical unbalanced diets. The inclusion of plant‐based foods also increases the content of some vitamins and minerals that are generally low in turkey. Bold font represents those that are ≥onefold increased after the substitution of plant foods. (AAFCO, 2019; USDA ARS NDL, 2015).

Abbreviations: AAFCO: Association of American Feed Control Officials, N/A: not available, IU: international unit.

^a^ Chloride and iodine content is not available in the USDA Food Composition Databases.

^b^ Vitamin A is calculated based on the estimated conversion efficiency in dogs (McDowell et al., 2000): 1 mg of β‐carotene or 2 mg of α‐carotene or 2 mg of β‐cryptoxanthin is equivalent to 833 IU of vitamin A; however, the NRC has not clearly defined a retinol equivalency for carotenoids. Vitamin D: 1 IU =0.025 μg cholecalciferol or ergocalciferol (Parker et al., 2017). Vitamin E: 1 IU =0.67 mg α‐tocopherol (NRC, 2006).

It is important to note that the AAFCO recommended minima are expressed in amounts per ME basis, with ME from modified Atwater factors or established through digestibility studies, while the food and nutrient databases only provide the amount of nutrients per ME calculated with Atwater factors (Normén et al., 2007; Rothwell et al., 2013; Touré & Xueming, 2010; USDA ARS NDL, 2015; Yang et al., 2019). The amount of nutrients are expressed on a caloric basis throughout this publication (unless only % dry matter was available and the conversion was not possible).

As previously discussed (Dillitzer et al., 2011), many home‐prepared and commercial diets comprising mainly animal‐based ingredients do not meet the minimal or recommended requirements for calcium, copper, zinc and vitamins A and D. The theoretical unbalanced diet in Table 2 of solely turkey meat and skin is consistent with findings from previous studies, showing that an adult dog would not meet its requirements for calcium (7% of the AAFCO requirement), potassium (74%), magnesium (87%), iron (70%), copper (33%), manganese (6%), zinc (80%), vitamins A (18%), D (58%), E (5%), B1 (50%) and folate (81%). However, when 10% kcal of turkey meat and skin is replaced by spinach, the amounts of calcium, potassium, magnesium, iron, copper, manganese, vitamins A, E and B1, and folate significantly increase. Similarly, substituting mushrooms for turkey meat and skin increases the amount of potassium, copper and vitamin B1 in the diet. Given that dogs have limited capacity to synthesize vitamin D from cholesterol (How et al., 1994), mushrooms are also a source of dietary vitamin D2 (ergocalciferol) (USDA ARS NDL, 2015), a form thought to be as biologically active as vitamin D3 (cholecalciferol) in dogs (Arnold & Elvehjem, 1939; Parker et al., 2017). Peas are also sources of magnesium, iron and vitamin A. The amounts of manganese and folate also increase with the inclusion of mushrooms, peas, blueberries or brown rice in the diet. Note that 10% of energy from a single plant‐based food was arbitrarily chosen as a theoretical example, and the imbalances that exist with such strategy should be highlighted. For example, a diet with 10% kcal from spinach may provide >1600% of the recommended minimum for folate, while it meets only 40% of the recommended minimum for calcium. A variety of ingredients, as well as fortification and supplementation as required, are key to a well‐balanced diet meeting the recommended allowances for adult dogs, and moreover, digestibility of nutrients must be considered.

## 4 ROLES OF PLANT‐BASED FOODS AND PHYTONUTRIENTS BEYOND MEETING NUTRIENT REQUIREMENTS

The overall effects of any diet are more complex than can be predicted by the essential nutrients only. There is increasing evidence that the inclusion of plant‐based ingredients and phytonutrients in the canine diet provide possible health benefits as described herein.

### 4.1 Body weight and body condition

Increased fibre intake is a common nutritional intervention used for weight management in dogs with an expansive amount of supporting literature; fibre has been found to improve body condition through multiple mechanisms (Baer et al., 1997; Butterwick et al., 1994; Silvio et al., 2000). While a previous review concluded that the results on fibre as an isolated strategy to promote weight loss appear mixed (Roudebush et al., 2008), this is likely due to differences of the design among studies. Given fibre is outside the scope of the present review, we instead focus on the ability of non‐fibre phytonutrients to promote weight control, for which the evidence is more limited.

The effects of soy isoflavones on weight loss and maintenance were reported in two published abstracts (Pan, 2006; Pan et al., 2008). Overweight dogs that received soy isoflavones were more likely to achieve the target weight on a weight loss diet (Pan et al., 2008). Similarly, for 12 months, a group of normal weight spayed/neutered Labrador Retrievers were fed diets in amounts 25% higher than their maintenance caloric requirements that were either supplemented with or without soy isoflavones. The group receiving no soy isoflavones gained twice as much body weight as the dogs in the other group (Pan, 2006). These findings may be due to the influence of soy isoflavones as oestrogen agonists in regulating energy metabolism and body condition, as previously observed in ovariectomized rats (Russell et al., 2017). This limited evidence suggests that it is probable that soy isoflavones can impact canine body condition through mechanisms requiring further exploration.

Obesity is a known cause of chronic inflammation (Loftus & Wakshlag, 2015). Green tea is rich in flavanols, notably catechin derivatives which have been associated with anti‐inflammatory and anti‐obesity properties (Hininger‐Favier et al., 2009; Li et al., 2020; Rahman et al., 2020; Yang et al., 2009). Sixteen healthy male Beagle dogs were fed a normal diet (dog chow; control), high‐fat diet or high‐fat diet supplemented with either a low dose of tea polyphenols (25%; 0.25 g/kg body weight) or a high dose (50%; 0.50 g/kg body weight) for 12 weeks (Rahman et al., 2020). The high‐fat diet induced obesity, and increased inflammation and hepatic weight. However, compared to consumption of a high‐fat diet, supplementation of tea polyphenols significantly reduced weight gain, reduced expression of inducible enzymes (inducible nitric oxide synthase (iNOS) and cyclooxygenase‐2 (COX‐2)) which are upregulated with inflammation, reduced mRNA expression of pro‐inflammatory cytokines (TNF‐α, IL‐1β and IL‐6) associated with liver inflammation, and lowered liver fat content (Rahman et al., 2020). These effects of the tea polyphenols were more pronounced with the higher dose. In another study, dogs fed a high‐fat diet for 18 weeks supplemented with green tea polyphenols (low (0.48%); medium (0.96%); and high (1.92%)) saw that polyphenol consumption similarly lessened weight gain, mitigated intestinal inflammation seen with the high‐fat diet, with reduction of pro‐inflammatory cytokines, and also served to counteract some of the resultant gut dysbiosis (Li et al., 2020). Collectively, these results demonstrate the anti‐obesity properties of tea polyphenols exhibiting clear impacts on weight management and obesity‐related inflammation in dogs using experimental models.

The effects of a nutritional supplement (4 g/kg body weight) on body composition and development were explored in growing Beagle puppies (Wang et al., 2017). Compared to the control group, the supplement offered increased amounts of vitamins A, C, E, D, B3 and B5, docosahexaenoic acid (DHA), taurine and carotenoids (β‐carotene, lutein and zeaxanthin). While the impact on body weight was inconclusive, puppies receiving the supplement showed significantly lower fat mass at six months of treatment. Those that received the supplement also displayed a healthier metabolic profile with improved serum triglyceride and cholesterol levels, and increased fat oxidation (Wang et al., 2017). Another study in growing Beagles offered a curcumin supplemented diet (1.5 mg/kg body weight) for 42 days (Campigotto et al., 2020). Curcumin is a polyphenolic compound derived from turmeric (*Curcuma longa*), with antioxidant properties (Chun et al., 2003). The supplemented diet did not impact body weight, and similarly, there were variable effects on inflammatory and immunological parameters, but total antioxidant capacity was increased. The authors note the results of curcumin on canine health were unclear (Campigotto et al., 2020). Curcumin has displayed low oral bioavailability, so novel forms may be required for increased absorption (Bolger et al., 2017). These studies indicate that early‐intervention with plant‐derived compounds are worth investigating further for promotion of weight management and health status in dogs.

Mannoheptulose, a naturally occurring sugar in alfalfa, avocado, fig and primrose, was investigated for its effect on body weight in energy expenditure in healthy adult Labrador Retrievers (McKnight et al., 2015). Mannoheptulose derived from unripened avocado flesh was provided to dogs at 6 mg/kg body weight in a double‐blind crossover trial. The mannoheptulose supplemented diet did not significantly impact energy expenditure and body weight compared to the control diet; however, dogs were found to have a lower fat to lean mass ratio on the treatment diet, and 3–5 h postprandial respiratory quotient was significantly decreased (McKnight et al., 2015). Mannoheptulose supplementation (8 mg/kg body weight) in healthy adult Beagles led to an increase in postprandial energy expenditure (5–10 h) (McKnight et al., 2014). Collectively, these results indicate that mannoheptulose supplementation may influence energy balance, but the associated impact on body weight and composition remains inconclusive.

### 4.2 Cardiovascular outcomes

The evidence on the cardiovascular benefits of plant foods and phytonutrients is emerging and discussed as follows. However, it is important to note that dogs are often experimentally manipulated to be used as a model to investigate the relationship between nutrition and cardiovascular health in humans, and many of these conditions may not naturally occur in dogs. Therefore, the clinical relevance of these studies in canine cardiovascular health remains unclear.

#### 4.2.1 Effects on serum lipids

While the risk of cardiovascular disease and its relationship to dietary fats or serum lipids has been relatively well established in humans, dietary fat does not have the same impact on blood lipid levels and cardiovascular disease risk in dogs. Dogs possess the ability to consume a high‐fat diet without risk of atherosclerosis given that most cholesterol is carried in high‐density lipoproteins in dogs (Boynosky & Stokking, 2014; Mahley et al., 1974). Rarely, however, serum cholesterol or triglycerides may require reduction due to secondary underlying health conditions (Boynosky & Stokking, 2014; Kagawa et al., 1998), which can possibly be combated with dietary modifications. Phytosterols are one of the dietary components demonstrating potential to benefit the canine cardiovascular system through cholesterol‐lowering properties.

Over the four‐week period of a randomized trial, serum total cholesterol of overweight and obese dogs fed either a dry black or navy bean powder diet (25% by weight) was decreased by 40 mg/dl (16%) and 54 mg/dl (23%), respectively, which was not significantly different from the decrease of 17 mg/dl (8%) observed in the control group consuming no beans. The effects of comparable weight loss identified in the groups on serum lipids cannot be ruled out (Forster et al., 2012). Serum triglyceride concentrations also decreased by 63 mg/dl (45%) in the navy bean powder group, but not the other two groups. Another trial in healthy dogs demonstrated a similar cholesterol‐lowering property of navy bean powder without concurrent weight loss (Forster et al., 2015). Cooked beans are a source of phytosterols, which were suspected to promote the lowered cholesterol levels as they share and compete for the same absorption pathway with cholesterol in the intestine, and thus, higher amount of cholesterol is excreted in the stool in the presence of phytosterols (Briand et al., 2006; Forster et al., 2012).

Astaxanthin, derived from *Haematococcus pluvialis*, was used in a trial to investigate its effect on serum lipids in healthy and obese Beagle dogs (Murai et al., 2019). Astaxanthin supplementation (0.3 mg/kg body weight/day) for 6 weeks in healthy dogs and 8 weeks in obese dogs significantly reduced plasma triglyceride concentrations, while there was no change in body weight. Markers of oxidative stress also decreased after astaxanthin supplementation, with its effect in obese dogs being more pronounced than healthy dogs.

#### 4.2.2 Effects on other cardiovascular measures

Elevated blood pressure is another known risk factor of cardiovascular diseases in dogs (Moses, 1954). A study evaluating cardiovascular measures in dogs after they gained comparable weight on either a lard (65% kcal from lard) or corn oil (65% kcal from corn oil) diet, found that dogs on the corn oil diet had a smaller increase in mean arterial pressure and a lower heart rate sensitivity to an adrenergic agonist (Truett et al., 1998). This study demonstrated the impact dietary fat composition can have on cardiovascular parameters in dogs, highlighting the differential effects of plant‐based fats, which are primarily unsaturated, in an admittedly extreme dietary intervention.

Studies have also been conducted to investigate the roles of phytochemicals on platelet aggregation, a known risk factor of thrombotic events. A flavonoid commonly found in various fruits and vegetables, quercetin, was administered at 50 mg/kg to dogs experiencing experimental myocardial infarction. Quercetin was found to be cardioprotective by helping to maintain heart function and circulation, limiting damage to tissues and coronary arteries, and preventing the formation of blood clots (Kolchin et al., 1991). Purple grapes have also been investigated due to their high content of phenolic compounds. Two studies in dogs with coronary stenosis demonstrated that purple grape juice (10 ml/kg body weight) reduced platelet aggregation and the subsequent cyclic flow reductions in coronary blood flow (Demrow et al., 1995; Osman et al., 1998), suggesting potential cardioprotective effects of flavonoids quercetin, kaempferol and myricetin found in purple grape juice. Additionally, grape seed and skin extracts, individually and in combination, were fed to healthy dogs at doses of 5 mg/kg body weight and 20 mg/kg body weight, respectively (Shanmuganayagam et al., 2002). Individually, neither of the extracts inhibited platelet aggregation, whereas in combination, as they would be in products such as grape juice, the antiplatelet effect was demonstrated (Shanmuganayagam et al., 2002). The administration of grape extracts to dogs, with known idiosyncratic renal damage from consumption of grapes (Martineau et al., 2016), would require further study. The impact of such studies employing the dog as an experimental model may be limited pending additional clinically relevant uses.

Atherosclerosis in humans is characterized by excessive reactive oxygen species (ROS) production that accelerates atherosclerotic plaque formation (Cervantes Gracia et al., 2017; Pignatelli et al., 2018). Dogs with congestive heart failure and dilated cardiomyopathy, while typically not having atherosclerosis, also have increased production of ROS and oxidative stress, alongside decreased levels of endogenous antioxidants (Freeman et al., 2005), although causality has not been established for these heart conditions. Endogenous antioxidative systems normally compensate for increased ROS production, during times of increased oxygen metabolism (Freeman, 1998). Some authors suggest that relative deficiency of endogenous antioxidant activity may impact cardiovascular diseases in dogs (Sagols & Priymenko, 2011). Therefore, supplying exogenous antioxidants through plant‐based phytonutrients in the diet may reduce ROS and oxidative stress, one of the proposed sequelae of cardiovascular diseases that affect dogs (Dunlap et al., 2006; Sagols & Priymenko, 2011).

#### 4.2.3 Effects on cardiovascular measures of exercise

The impact of antioxidant administration in exercise has received limited attention. After 18 weeks of supplementation, an antioxidant formulation containing grape seed extract, quercetin, blueberry, resveratrol, and strawberry and blackberry dried extracts was demonstrated in healthy therapy dogs to significantly reduce exercise‐induced oxidative stress by neutralizing excess ROS production (Sechi et al., 2017). The antioxidant properties of blueberries were further shown to attenuate post‐exercise oxidative damage and elevate antioxidant status in healthy sled dogs who were supplemented with approximately 20 g of blueberries for four days (Dunlap et al., 2006). Likewise, dogs provided 400 IU of α‐tocopherol, 3 mg of β‐carotene and 20 mg of lutein daily for one month showed increased plasma antioxidant concentrations and lipoprotein resistance to oxidation, as well as decreased DNA oxidative damage induced by a bout of exercise (Baskin et al., 2000). These studies collectively show the ability of antioxidants to reduce exercise‐induced oxidative stress which could be hypothesized to apply to other instances of oxidative stress in naturally occurring canine cardiovascular diseases but more clinically relevant data are required for confirmation.

### 4.3 Insulin sensitivity and glycaemic control

Insulin sensitivity refers to the ability of endogenous insulin to decrease blood glucose by stimulating peripheral consumption and reducing glycogenolysis to maintain euglycaemia. Insulin resistance is often imprecisely defined but is broadly associated with metabolic dysfunction even in non‐diabetic dogs (Xenoulis et al., 2011). Many studies have investigated the effect of macronutrient composition, dietary fibres, food restriction, exercise, and weight loss on insulinemic and glycaemic controls in both healthy and diabetic dogs. Previous reviews explore these topics in greater detail (de Godoy et al., 2013; Ihle, 1995; Rankovic et al., 2020; Wynn, 2001). However, novel plant‐based and herbal ingredients with potential insulinemic and glycaemic effects are reviewed in this section.

Texturized soy protein is used as a protein source in some commercial dog foods. When a group of healthy dogs were fed a high‐fat diet, replacing beef with texturized soy protein led to a decrease of insulin secretion during the first 2–3 h after the meal (Hill et al., 2006). The authors discussed that this effect may have originated from the carbohydrate and fibre content of the soy, and not the protein, particularly via the impact on slowing the rate of nutrient absorption in the intestine. Indeed, glycaemic response has been shown to vary when different grains and pulses were used as a carbohydrate source in extruded diets (Adolphe et al., 2015; Carciofi et al.,l., 2008; Quilliam et al., 2021; Rankovic et al.,l., 2020; Teixeira et al.,l., 2018). The degree of fatty acid saturation may also have a differential impact on insulin sensitivity. Dogs were induced to gain comparable weight on either a lard (65% kcal from lard) or corn oil diet (65% kcal from corn oil) (Truett et al., 1998). It was found that only dogs on the lard diet developed some insulin resistance, while weight gain from the corn oil diet did not affect any insulin sensitivity.

Some of the plants used for medicinal purposes have been investigated in non‐diabetic mongrel dogs. Annatto, derived from the seeds of the achiote tree (*Bixa orellana*), is commonly used as a food colouring agent due to its carotenoid content. Annatto at 80 mg/kg body weight was demonstrated to suppress the postprandial rise in blood glucose level and increase plasma insulin level after an oral glucose load (Russell et al., 2005). Increased insulin affinity at the receptor was also observed on mononuclear leukocytes and red blood cells. Similarly, capsaicin was extracted from bird peppers (*Capsicum frutescens*); 9 mg of capsaicin administered intravenously led to a reduced blood glucose and an increased insulin level during the oral glucose tolerance test (Tolan et al., 2001), but this impact still needs to be evaluated with oral administration.

Rosemary (*Rosmarinus officinalis*) and basil (*Ocimum basilicum*) were recently studied in Rottweiler dogs (Abdelrahman et al., 2020). Dietary fortification with rosemary leaves or basil leaves separately at 0.05%, or in combination at 0.025% each, significantly reduced fasting glucose level as compared to the control group after two months on each diet. The hypoglycaemic effect was correlated with the increased insulin secretion in those receiving basil leaves alone and those receiving the combined fortification. The authors attributed the polyphenol content of rosemary and basil to their hypoglycaemic effect and positive impact on pancreatic β‐cell function.

Green tea is a rich source of flavonols including EGCG. In one study, a group of obese Beagles were randomly assigned to receive a green tea extract (80 mg/kg body weight) or a placebo for 12 weeks (Serisier et al., 2008). The green tea extract contained 35.7 mg/g of epicatechin, 64.8 mg/g of epicatechin gallate, 20.2 mg/g of epigallocatechin and 153.1 mg/g of EGCG. Insulin sensitivity index significantly improved only in the green tea group by 60%, even when body weight and fat mass did not change. Expression of genes related to glucose and lipid metabolism including PPARγ, LPL, adiponectin, and GLUT4 mRNA in visceral and subcutaneous adipose tissues elevated with the green tea supplementation. In skeletal muscle, the expression of PPARα and LPL also increased with green tea.

Mannoheptulose was investigated as a novel functional food ingredient for its glycolytic inhibiting property (McKnight et al., 2018). A group of healthy Labrador Retriever dogs were fed mannoheptulose (6 mg/kg body weight) in a crossover trial with the use of isotope labelling technique. However, this dietary dose of mannoheptulose showed no effects of glucose turnover and oxidation, lipolysis rate, and fatty acid oxidation.

### 4.4 GI health and the gut microbiota

The GI tract plays a critical role in nutrient digestion and absorption, which in part explains its susceptibility to dietary interventions. The canine digestive tract is further a host environment for many microorganisms, notably bacteria, comprising the microbiome, which is unique to each individual dog (Garcia‐Mazcorro et al., 2017). Possessing a healthy microbiome and maintaining gut barrier integrity are important for canine health and disease prevention. Many plant components influence the microbiota by increasing potentially beneficial bacteria and minimizing pathogenic microbes (Kil & Swanson, 2011; Pinna & Biagi, 2014; Suchodolski, 2011a, 2011b,2011a, 2011b). To illustrate, dogs with inflammatory bowel disease, whose clinical symptoms are commonly characterized by chronic vomiting and diarrhoea, were revealed to have decreased abundance of *Firmicutes* and *Bacteroidetes*, as well as lower gut microbial diversity, specifically in the genus *Clostridium* (Honneffer et al., 2014; Minamoto et al., 2015). Substances which can promote selected bacterial growth and alter the microbiome structure, benefiting the host's health, are commonly known as prebiotics (Garcia‐Mazcorro et al., 2017). While traditionally prebiotics were considered to be subsets of fermentable fibres (Garcia‐Mazcorro et al., 2017), the definition has since broadened to encompass a wide variety of ingredients (Gibson et al., 2010, 2017), including non‐carbohydrate plant‐based ingredients, notably polyphenols (Gibson et al., 2017; Moorthy et al., 2020). Prebiotics are also known to influence short‐chain fatty acid (SCFA) populations including acetate, propionate and butyrate, which serve as an energy source for colonocytes in the large intestine, with butyrate serving as the preferred energy source (den Besten et al., 2013; Minamoto et al., 2019; Ríos‐Covián et al., 2016; Sunvold et al., 1995). The prebiotic effects of fibres on the canine microbiome have been previously and extensively reviewed (Pilla & Suchodolski, 2021; Pinna & Biagi, 2014; Wernimont et al., 2020; Ziese & Suchodolski, 2021).

Grape proanthocyanidins were demonstrated to have varying effects on the faecal microbiota of healthy adult dogs fed a standardized diet. After 28 days, supplementation with 1 mg/kg body weight grape proanthocyanidins significantly increased the abundance of *Escherichia* and *Eubacterium*, while 3 mg/kg body weight increased relative abundances of *Fusobacterium* and *Phascolarctobacterium* (Scarsella et al., 2020). However, dogs receiving a placebo also saw altered abundances of select faecal microbiota populations, which supports the notion that microbiome communities and responses to prebiotics are variable among individuals. Significant increases in faecal propionate were also found with proanthocyanidins supplementation (3 mg/kg body weight); however, increased isobutyrate concentrations were seen with the placebo tablets (Scarsella et al., 2020). Polyphenol supplementation from pomegranate peel extract (50 mg/kg body weight) was found to increase faecal concentrations of total SCFAs and fermentative metabolites, as well as improve antioxidant status in healthy dogs (Jose et al., 2017). Additionally, supplementation with green tea polyphenols was shown to alter the structure of the gut microbiota in 30 adult male dogs after 18 weeks (Li et al., 2020). Compared to dogs consuming a high‐fat diet, supplementation with green tea polyphenols (low (0.48%); medium (0.96%); and high (1.92%)) attenuated the reduction in α‐diversity indices seen with the high‐fat diet. Additionally, polyphenol consumption altered the relative abundances of faecal microbiota populations, decreasing *Bacteroidetes* and *Fusobacteria* and increasing *Firmicutes* (Li et al., 2020). These studies are among the first to explore the use of polyphenols as prebiotics in dogs; however, reviews of both animal (Moorthy et al., 2021) and human studies (Moorthy et al., 2020) further support the growing body of evidence highlighting the ability of polyphenols to function in this capacity.

Supplementation with green tea polyphenols also diminished some of the markers of intestinal inflammation seen in dogs on the high‐fat diet, which may be an important consideration in the avoidance of canine enteropathies (Li et al., 2020). Since both chronic and acute enteropathies have been associated with increased oxidative stress and decreased antioxidant capacity in dogs (Candellone et al., 2020; Minamoto et al., 2015; Rubio et al., 2017), the antioxidant and bactericidal properties of select plant‐based phytonutrients have been of increasing interest as a possible therapeutic measure. Candellone et al., 2020 discuss the potential of polyphenol supplementation for management of acute diarrhoea in dogs due to their anti‐inflammatory, antioxidant, and antimicrobial properties and their reciprocal relationship with the gut microbiome. The authors appropriately state the clear need for more robust clinical studies to confirm polyphenols as a treatment option or possible complementary approach to traditional therapies used for management of canine acute diarrhoea and inflammation (Candellone et al., 2020).

### 4.5 Immune health

Maintenance of a normal immune system is critical for optimizing health, and abnormalities in immune function can be associated with significant morbidity. Food allergies are immune‐mediated in dogs and linked to dermatological and GI signs (Verlinden et al., 2006). Additionally, a compromised immune system can make dogs more susceptible to infections, immune‐mediated disease and neoplasia, while certain cancers may be inherently immunosuppressive in dogs (Bergman, 2009; Elwood & Garden, 1999; Mucha et al., 2016; Tizard, 2000). Cancer is extremely common in aged dogs, appearing in approximately 1 in every 3–4 dogs, and it is one of the leading causes of death (Dobson et al., 2002; Merlo et al., 2008). This section lists several plant‐based compounds or ingredients that possess possible immune‐modulating effects in dogs.

Studies have attempted to identify specific immunological functions of individual compounds. Vitamin C supplementation at 60 mg/d increased blood CD4+ T cells by approximately 10% in healthy dogs, but it did not appear to have an impact on other circulating lymphocytes or immunoglobulins (Hesta et al., 2009). Carotenoids were demonstrated in at least three studies to have immune‐modulating effects. After Beagle dogs were fed a daily dose of 0, 2, 20 or 50 mg of β‐carotene for eight weeks, plasma antibody immunoglobulin G concentrations increased in a dose‐dependent fashion (Chew, Park, Weng, et al., 2000; Chew, Park, Wong, et al., 2000). Additionally, dogs provided 20 or 50 mg β‐carotene showed higher CD4+ T‐cell levels and displayed augmented delayed‐type hypersensitivity (DTH) response to both specific and non‐specific immune response triggers (Chew, Park, Weng, et al., 2000; Chew, Park, Wong, et al., 2000). Similarly, enhanced DTH to immune response triggers has also been demonstrated in female Beagle dogs supplemented with 0, 5, 10 or 20 mg lutein daily for 12 weeks (Kim et al., 2000). Lutein consumption also increased immunoglobulin G production and the population of lymphocyte subsets CD4+ T cells, CD5+ T cells, CD8+ T cells and MHC class II+lymphocytes (Kim et al., 2000). Consequently, both β‐carotene and lutein have demonstrated the ability to enhance cell‐mediated and humoral immune response in female Beagle dogs (Chew, Park, Weng, et al., 2000; Chew, Park, Wong, et al., 2000; Kim et al., 2000). Additionally, a study in a group of older dogs (mean age 10.6 years) demonstrates that supplementation with β‐carotene at 17.9 mg/kg diet improved immunological variables by increasing levels of CD4+ T cells, improving T‐cell proliferation and heightening DTH responses (Massimino et al., 2003). The long‐term clinical effects of these immunological shifts remain unknown.

Plant‐based ingredients with antioxidant properties are also being examined for immune‐modulating properties. Both *Echinacea angustifolia* (0.10 mg/kg body weight of echinacoside) and *Vaccinium myrtillus* extracts (0.20 mg/kg body weight of anthocyanidin) downregulated the expression of inflammatory genes in circulating white blood cells in healthy dogs after 60 days (Sgorlon et al., 2016). The combined effects of dietary antioxidants and behaviour enrichment on inflammatory and immune response of healthy, geriatric Beagles were tested in a two‐year trial (Hall et al., 2006). Antioxidant enriched food included vitamin E (1000 ppm), L‐carnitine (275 ppm), lipoic acid (125 ppm), vitamin C (80 ppm), fruits and vegetables (spinach flakes, tomato pomace, grape pomace, carrots and citrus pulp, each included at 1%) (Hall et al., 2006). Inflammatory and immune cells were analysed *ex vivo* by stimulating and assessing peripheral blood neutrophils for phagocytosis and peripheral blood lymphocytes for steroid‐induced apoptosis and phenotypic markers. This study indicated that behavioural and antioxidant enrichment in combination served to increase neutrophil phagocytosis and B‐cell populations. However, antioxidant supplementation alone did not prove statistically effective without the added behavioural intervention (Hall et al., 2006).

Finally, the correlation between developing transitional cell carcinoma (TCC) of the bladder and dietary vegetable consumption was described in 175 Scottish Terriers in a retrospective case‐control study. Scottish Terriers are among the breeds known to be at higher risk of TCC and may serve as an animal model for human TCC (Hayes, 1976; Knapp et al., 2014). An inverse relationship with consumption of any vegetables (as a part of commercial diets, home‐prepared diets, and table scraps) at least three times per week and the risk of developing TCC was observed (OR = 0.44, 95% CI = 0.23–0.83) (Raghavan et al., 2005). Yellow‐orange vegetables and green leafy vegetables specifically were significantly associated with a 61% (OR = 0.39, 95% CI = 0.19–0.82) and 90% (OR = 0.10, 95% CI = 0.01–0.84) decrease in risk of developing TCC, respectively, which is accredited to their high content of carotenoids. However, dietary fibre, phytosterols and phenols are phytonutrients and anti‐carcinogenic compounds present in vegetables, which could prevent TCC development. Results of this retrospective study that includes a clinical end point provide positive indications for vegetable consumption for canine cancer prevention albeit in a narrow cohort. Collectively, the studies in this section have demonstrated that inclusion of a wide variety of plant‐based ingredients can differentially alter the canine immune system, and continued research will help to further interpret the clinical relevance.

### 4.6 Bone and joint health

Musculoskeletal disorders occur in 24% of all dogs (Johnson et al., 1994). The most prevalent musculoskeletal disorder, osteoarthritis (OA), is one of the main causes of lameness and disability and estimated to affect 20% of all dogs over one year of age (Bennet, 1990; Brown, 2017; Budsberg & Bartges, 2006). Other common musculoskeletal issues found in dogs include osteochondrosis and canine hip dysplasia, which are further suspected to be related to nutrition, providing possibilities for nutritional interventions (Richardson & Zentek, 1998). While many nutritional causes may be linked to calcium, cholecalciferol and other non‐plant‐based ingredients, certain phytonutrients and plant‐based constituents may exert beneficial effects in the management of orthopaedic disorders.

Avocado/soya bean unsaponifiables (ASU) are vegetable extracts composed solely of the total fraction of unsaponifiables from one‐third avocado oil and two‐thirds soya bean oil (Boileau et al., 2009). They belong to a group of pharmacological agents or nutritional supplements called symptomatic slow‐acting drugs for OA, which are known to aid in the long‐term effects of OA in humans (Dougados, 2006). Thus, they were examined in a clinical trial for their ability to affect structural changes in the early developmental stages of OA in dogs. Osteoarthritis was experimentally induced by transection of the cranial cruciate ligament, and ASU (10 mg/kg body weight daily) were shown to reduce osteoarthritic structural changes over an eight week study period by limiting subchondral bone remodelling and slowing cartilage breakdown (Boileau et al., 2009). In another trial, it was demonstrated that ASU supplementation increased cytokine TGF‐β_1_ and TGF‐β_2_ levels in the synovial fluid of healthy dogs (Altinel et al., 2007). Both TGF‐β isoforms are expressed by chondrocytes and osteoblasts to stimulate cartilage matrix synthesis (Boumediene et al., 1999), which may explain reduced breakdown of cartilage observed in the aforementioned trial (Boileau et al., 2009). Additionally, ASU were shown to inhibit the inflammatory effect of IL‐1β and stimulate collagen synthesis (Mauviel et al., 1989, 1991), as well as to inhibit the production of the inflammatory cytokines IL‐6, IL‐8 and eicosanoid prostaglandin E2 in articular chondrocytes (Henrotin et al., 1998). Collectively, these studies illustrate the potential of ASU to benefit canine joint health by protecting against cartilage degradation and damaging inflammation.

OA is associated with chronic inflammation and free radical production; the polyphenolic compound curcumin has been studied for its ability to ameliorate these changes. Curcumin possesses the ability to scavenge free radicals and target inflammatory responses, specifically via inhibition of several inflammatory mediators (Chun et al., 2003; Singh & Aggarwal, 1995; Surh et al., 2001). Transcriptomics of peripheral white blood cells in dogs with OA were measured before and after curcumin supplementation (4 mg/kg body weight, twice a day) for 20 days, and changes in the expression of genes related to inflammation and connective tissue development and function were observed (Colitti et al., 2012). In a similar study, supplementation of turmeric extract (6.60 mg/kg body weight of curcumin) for 60 days also led to a downregulation of inflammatory genes in circulating white blood cells in dogs with a history of OA (Sgorlon et al., 2016). Additionally, P54FP, an extract of turmeric, *C*. *domestica* and *C*. *xanthorrhiza*, contains polyphenolic phytochemicals (curcumoids) and essential fatty acids (Budsberg & Bartges, 2006). Use of P54FP for treatment of canine elbow or hip OA was investigated in a controlled trial for eight weeks, but the results were inconclusive as the overall improvement was observed only when it was assessed by the study investigators but not the owners (Budsberg & Bartges, 2006; Innes et al., 2003). Other plant‐based ingredients such as the resin extract of the tree *Boswellia serrata* may also have promise as dietary supplements (Miscioscia et al., 2019). A *B*. *serrata* extract was investigated in an open‐label multicentre trial including 25 dogs with confirmed OA (Reichling et al., 2004). After six weeks of supplementation, significant improvements were observed for intermittent lameness, pain and gait, although no control group was included in the study. Cannabidiol, a non‐psychoactive cannabinoid found in hemp and marijuana, despite not being GRAS, is of increasing interest for a wide variety of medical applications in dogs, notably for treatment of OA (Gamble et al., 2018). Recent studies are demonstrating the tolerability of cannabidiol for dogs (Bartner et al., 2018), as well as its ability to significantly relieve pain associated with OA (Gamble et al., 2018).

Vitamin C, or ascorbic acid, is naturally found in a variety of fresh fruits and vegetables but is also added to some commercial pet foods. Vitamin C is a cofactor for lysyl (EC 1.14.11.4) and prolyl hydroxylases (EC 1.14.11.7 and EC 1.14.11.12) (Pinnell, 1985), which are essential for the biosynthesis of collagen (Richardson & Zentek, 1998). Growing puppies and adult dogs do not have a dietary requirement for vitamin C, as they have the ability to synthesize it endogenously from other substrates (McDowell, 2000; Richardson & Zentek, 1998), but short‐term supplementation (on average 5.2 mg/kg body weight, for 36 days) was shown to raise its serum concentration (Hesta et al., 2009). When dogs with early stages of hypertrophic osteodystrophy, a developmental bone disease mainly affecting large breeds, were treated with oral vitamin C (40 mg/kg body weight, daily) and injection of sodium salt of hyaluronic acid (20 mg, 5× every 10 days) for 60 days, decreased inflammation and pain around joints, enhanced collagen synthesis, and the disappearance of radiological features of hypertrophic osteodystrophy were observed (Aleksiewicz et al., 2013). Treatment effects may be mediated by an increase of bone mineralization as observed by reduced serum calcium and phosphorus concentrations and lowered alkaline phosphatase activity (Aleksiewicz et al., 2013). However, the effects of vitamin C alone could not be independently evaluated, and no control group was present for comparison.

The carotenoid lycopene, found mostly in red fruits such as watermelons and tomatoes (Table 1), and flavonoid baicalein, found in selected plants such as thyme, were both individually examined for their effects on canine osteosarcoma cell lines, a common canine neoplasm (Helmerick et al., 2014; Wakshlag & Balkman, 2010). Osteosarcoma accounts for 5% of all canine tumours and, due to its prevalence and poor long‐term prognosis, requires additional therapeutic options (Helmerick et al., 2014; O’Donoghue et al., 2010). Treatment of canine osteosarcoma cells with either lycopene or baicalein reduced cell proliferation and induced apoptosis of different cell lines to varying degrees (Helmerick et al., 2014; Wakshlag & Balkman, 2010). Similarly, the flavonoid myricetin was also shown to induce apoptosis in canine osteosarcoma cells (Park et al., 2018). Future clinical studies are still required to establish dietary doses and any *in vivo* anti‐neoplastic properties of these compounds.

An intervention with a combination of nutrients was examined in a few trials. A diet fortified with vitamins C and E, β‐carotene, mitochondrial cofactors, eicosapentaenoic acid (EPA) and DHA was able to improve the joint mobility parameters of the shoulder in aged dogs after six months as compared to the basal diet (Lorke et al., 2020). In another randomized controlled trial, a dietary supplement consisting of curcuminoids, hydrolysed collagen and green tea extract was examined in dogs with signs of OA for three months (Comblain et al., 2017). When evaluated by veterinarians, a significant reduction of pain during manipulation was observed in the supplement group but not the control group, whereas other parameters showed no difference between groups. Owner‐assessed pain severity score also remained stable in the supplement group while it worsened in the control group. Altogether, several plant‐based ingredients, especially those containing polyphenolic compounds and carotenoids, appear particularly relevant targets for more robust clinical trials in the management of canine bone and joint health.

### 4.7 Renal Health

Renal disease is relatively common among dogs, affecting approximately 1% of all dogs, with 15–20% of older dogs experiencing some form of renal injury (Brown et al., 1998; Burkholder, 2000). Sustained oxidative damage is believed to play a role in the progression of canine renal disease, contributing to variable renal interstitial fibrosis, glomerulosclerosis and hypertension (Brown, 2008; Elliott, 2006; Locatelli et al., 2003). Plant‐based antioxidants have therefore been hypothesized to be beneficial in combination with standard dietary therapy (e.g. phosphorus restriction) for the management of chronic kidney disease.

Prospective controlled trials have evaluated the impact of functional foods, including plant‐based foods and phytonutrients, on renal function measures such as glomerular filtration rate, serum levels of symmetric dimethylarginine (SDMA) and creatinine, and urinary nitrogenous wastes (Case et al., 2011). Non‐enzymatic antioxidants, including carotenoids, flavonoids and polyphenols, may reduce renal oxidative stress (Brown, 2008). In Beagles, whose ages ranged from 6 to 8 years, 16‐month supplementation with vitamin E and carotenoids reduced proteinuria, glomerulosclerosis and interstitial fibrosis independent of the omega‐3:omega‐6 fatty acid ratio in the diet (Brown, 2008). Ongoing studies are further analysing the use of antioxidants in dogs, including appropriate dose, but this limited evidence supports inclusion of higher antioxidant levels as an adjunct in efforts to prevent further damage to diseased kidneys.

Two hundred and ten geriatric dogs were fed either a diet of owner's choice or a test diet supplemented with fish oil, antioxidants (lipoic acid, vitamins C and E), L‐carnitine, increased amounts of fruits and vegetables (beet pulp, citrus pulp, carrot granules, dried spinach and tomato pomace), bioavailable protein and reduced sodium (Hall, MacLeay, et al., 2016; Hall, Yerramilli, et al., 2016). Dogs receiving the test diet showed significantly decreased levels of SDMA and creatinine over the six‐month period. A subgroup analysis in dogs meeting the criteria of IRIS Stage 1 CKD also highlighted the use of nutritional interventions, including plant‐based ingredients, to sustain and improve canine renal function in nonazotemic dogs with elevated SDMA. In another randomized trial, a group of geriatric dogs that demonstrated lower renal function as compared to younger dogs were fed a traditional renal protective food diet with or without varying amount of functional foods (Hall, MacLeay, et al., 2016; Hall, Yerramilli, et al., 2016). The traditional renal protective food diet was energy dense with protein and phosphorus restriction, and the functional foods were fish oil, antioxidants, fruits and vegetables, and protein from egg and chicken meat versus other protein sources in the control diet. After six months, the glomerular filtration rate was shown to improve in all dietary groups, but serum SDMA concentrations only decreased in those supplemented with functional foods. These studies suggest an opportunity for plant‐based ingredients to aid in the management of canine renal disease, but long‐term studies with only one nutritional variant are required to identify clinical relevance.

### 4.8 Skin and coat health

Dog owners are often concerned with the appearance of their dog's skin and coat, in part because of the assumption that coat appearance is a gauge of canine health status (Lloyd & Marsh, 1999). The skin is a very large metabolically active organ that has a high requirement for protein, fatty acids, and several vitamins and minerals (Watson, 1998). Skin disorders in dogs rarely result from nutrient deficiencies (nutritional dermatosis), but can be due to absorption issues, comorbidities, reduced intake, genetic factors or malnutrition. A recent review listed some nutritional supplements that may have an impact on canine dermatological disorders (Marchegiani et al., 2020). Several plant‐based ingredients, especially plant oils and those with antioxidant properties, are gaining recognition for their benefits to skin and coat health.

Essential fatty acids for dogs include omega‐6 and omega‐3 polyunsaturated fatty acids (Bauer, 2016). Signs of fatty acid deficiency in dogs include dry and dull coat, hair loss, and rarely, pruritus (Lloyd & Marsh, 1999). Linoleic acid (LA) is an omega‐6 polyunsaturated fatty acid with an AAFCO minimum dietary requirement of 2.8 g/1000 kcal diet (Table 2). LA contributes to the cutaneous water permeability barrier, which prevents evaporative water loss (Watson, 1998). A major source of LA are vegetable oils, such as sunflower or safflower oil (USDA ARS NDL, 2015). On the contrary, omega‐3 fatty acids EPA and DHA are primarily found in marine sources (fish and algae) and have shown beneficial effects in atopic dermatitis and other skin disorders. Dogs have the ability to convert the plant‐based omega‐3 fatty acid ɑ‐linolenic acid (ALA) to EPA and DHA endogenously, although the conversion rate is relatively low (Dunbar et al., 2010). When 18 healthy dogs were supplemented with either flax seeds (29% LA, 10% ALA) or sunflower seeds (37% LA, 2% ALA) in a prospective study (no control group), improvements in skin and hair coat condition scores, as evaluated by six blinded evaluators (2 nutritionists, 2 dermatologists and 2 laboratory technicians), were indicated after 28 days in both groups (Rees et al., 2001). However, continued improvements were not noted to the end of the study on day 84. It is hypothesized that improvements in skin and coat condition were associated with increasing levels of plasma polyunsaturated fatty acids whose high levels had already been achieved after one month, and thus, continued improvements in condition scores were not seen (Rees et al., 2001). Similarly, it was demonstrated in a randomized controlled trial that supplementation of zinc (100 mg/1000 kcal) and LA (15.1 g/1000 kcal) to a complete and balanced diet significantly improved coat condition in healthy adult dogs (Marsh et al., 2000). This supplemented diet also reduced the transepidermal water loss over time.

In dogs suffering from atopic dermatitis (AD), a common inflammatory allergic skin disease, reported to affect about 10% of dogs (Noli et al., 2007), plant‐based essential fatty acids are also proving therapeutic. A randomized controlled trial demonstrated the therapeutic benefit in a group of 50 dogs with AD. The dogs receiving the diet with the highest LA (23% of total fatty acids or 6.6 g/1000 kcal) and ALA (2.2% of total fatty acids or 0.6 g/1000 kcal) content had the best coat appearance assessed by their owners at the end of the study (Glos et al., 2008), although this diet also contained significant amounts of EPA and DHA from fish. Another study provided 24 dogs with AD polyunsaturated fatty acids from black currant seed oil (BSO) (90 mg/kg body weight), conjugated linoleic acid (90 mg/kg body weight) or both (90 mg/kg body weight each) for two months (Noli et al., 2007). None of the interventions yielded significant results; however, the best clinical improvements in AD were seen with BSO provided alone. BSO is known for anti‐inflammatory and antioxidative effects and is rich in omega‐3 fatty acids and omega‐6 fatty acid γ‐linolenic acid (GLA) (Bakowska‐Barczak et al., 2009), while conjugated linoleic acid is thought to benefit immunological and allergic conditions and contains geometric isomers of LA (Noli et al., 2007). BSO‐treated dogs did have significantly higher serum levels of fatty acids GLA and dihomo‐γ‐linolenic acid, which serves as a substrate for anti‐inflammatory eicosanoids such as prostaglandin E1 and 15‐hydroxyeicosatrienoic acid (Noli et al., 2007). Additionally, BSO antioxidative properties are attributed to its high concentration of polyphenolic compounds, notably phenolic acids and flavonols, alongside its tocopherol and phytosterol content (Bakowska‐Barczak et al., 2009). Borage seed oil, a potent source of GLA, was given in combination with fish oil to 21 dogs with canine atopy for eight weeks in one of the following doses per kg body weight: 176 mg borage oil +22 mg fish oil, 88 mg borage oil +11 mg fish oil +102 mg olive oil or 204 mg olive oil (Harvey, 1999). At the conclusion of eight weeks, dogs receiving borage oil and fish oil in both groups showed significantly improved total measures of atopy (pruritus, erythema, oedema, alopecia and self‐excoriation). While the isolated effects of borage seed oil on canine atopy were not examined, this study indicated that providing high levels of GLA in combination with fish oil does produce anti‐inflammatory activity and improve the skin condition of dogs (Harvey, 1999). Dogs provided a plant‐based supplement containing thyme (*Thymus vulgaris*), rosemary (*R*. *officinalis*), lemon balm (*Melissa officinalis*), absinthe (*Artemisia absinthium L*.), lemongrass (*Cymbopogon citratus*) and fenugreek (*Trigonella foenum G*.) for 150 days had significantly decreased flea populations compared to dogs receiving a placebo by day 30 (Moog et al., 2020). Given the prevalence of flea allergy dermatitis in dogs (Lam & Yu, 2009), this study demonstrated how the use of plant‐derived ingredients may be useful for preventing or controlling the pruritic skin disease albeit not likely to a level of complete flea reduction necessary to ameliorate clinical signs.

Vitamin E is a group of eight fat‐soluble isoforms of tocopherols and tocotrienols that are commonly found in plant oils, nuts and seeds. While antioxidants, such as vitamin E, are frequently added to foods, as a preservative to prevent oxidation, and thereby helping to increase shelf life and maintain nutrient content (Wilson et al., 2017), they also have many crucial cellular roles, including those in dermatology (Keen & Hassan, 2016). Vitamin E is a dietary fat‐soluble antioxidant that is concentrated in the lipid‐soluble portion of the cell membrane (Keen & Hassan, 2016; Thiele et al., 1999). It is also a constituent of sebum that is constantly secreted to the surface of the skin and, being a strong antioxidant, is proposed to reduce oxidative damage from the environment such as the UV rays that generate free radicals (Nachbar & Korting, 1995; Thiele et al., 1999). While it is unclear if this photoprotective effect occurs to the same degree in dogs due to their fur, it has been shown that dogs with shorter coats and lighter skin are more susceptible to UV damage, and that increased consumption of vitamin E does lead to increased serum and cutaneous levels in dogs, and decreases some biomarkers of oxidative stress (Jewell et al., 2002). Further, vitamin E status is correlated with the risk of AD because lower plasma levels of tocopherols were observed in dogs with such conditions (Plevnik Kapun et al., 2013). In one trial, 29 dogs with AD were supplemented with vitamin E oil (8.1 IU/kg body weight) or placebo (mineral oil) daily for eight weeks. Dogs receiving vitamin E had significantly higher plasma total antioxidant capacity and reduced severity of AD at the end of the study (Plevnik Kapun et al., 2014).

Soy was historically a common ingredient in dog food. Due to their oestrogenic activity at high doses, soy isoflavones were suspected to impact the hormonal status of dogs and cause negative impacts to skin quality, along with hair loss and thinning (Cerundolo et al., 2009). However, in a prospective controlled randomized trial, 30 normal dogs were either fed a high‐ or a low‐isoflavone soy‐based diet for 12 months. Upon conclusion of the study, no detectable differences were found in skin and coat quality, and the authors concluded that inclusion of soy isoflavones appears to have no positive or negative impact on skin health (Cerundolo et al., 2009).

Finally, in a pre‐clinical study, a nutrient combination of aloe vera, curcumin, taurine and vitamin C was investigated for its effect on maintaining the canine skin barrier (Fray et al., 2004). This *in vitro* study demonstrated the ability of this nutrient combination to increase migration of canine fibroblasts and limit the diffusion of water through canine keratinocyte cells, both of which are crucial to skin barrier function (Fray et al., 2004). Likewise, another recent *in vitro* study showed that a nutraceutical mixture of luteolin, piceatannol and cannabidiol increased DNA methylation at specific gene regulatory regions and downregulated the expression of inflammatory genes in inflamed canine keratinocytes and monocytes (Massimini et al., 2021). These studies show that plant‐based foods, notably seed oils and those containing polyphenolic compounds, may have clinical relevance in maintenance of the canine skin and coat.

### 4.9 Visual health

Dogs, like humans, experience age‐related eye diseases. In dogs, these may include lenticular sclerosis (also known as nuclear sclerosis) and progressive retinal atrophy. Lenticular sclerosis affects >50% of dogs over 10 years of age, but does not significantly compromise visual function (Williams et al., 2004), and therefore, no medical treatment is often recommended. Only severe cases of lenticular sclerosis may cause a refractive error or myopic shifting, or short sightedness (images from a far distance appear blurry) (Miller & Murphy, 1995; Murphy et al., 1992). Progressive retinal atrophy is a common form of canine retinal degenerative disease in many breeds that can cause total blindness in advanced stages (Bunel et al., 2019). Currently, there is no medical treatment for progressive retinal atrophy, and the cause is unknown but may be related to increased oxidative stress in the retinal pigment epithelium (Biswal et al., 2016).

Numerous studies have evaluated antioxidants in human ocular function, especially for carotenoids lutein and zeaxanthin (Johnson, 2014), but very limited evidence exists for the canine eye. A randomized controlled trial in 12 Beagles with healthy eyes demonstrated that a daily dose of 20 mg lutein, 5 mg zeaxanthin, 20 mg β‐carotene, 5 mg astaxanthin, 180 mg vitamin C and 336 mg vitamin E for six months significantly improved retinal responses in both dark and light conditions, which likely reflects a delay of retinal degeneration in ageing (Wang et al., 2016). This effect may vary among dog breeds based on inherent differences in ageing (Rosolen et al., 2010). Moreover, while refractive error changes over the six‐month study period were observed in the control group, the refractive error changes were reduced in the group fed the supplement (Wang et al., 2016). It is still unclear if the observed decline in aged dogs affects their function or is readily apparent to their owners. Despite the control group in this study being fed a diet meeting the AAFCO requirements, adding non‐essential nutrients (carotenoids and vitamins C) as well as vitamin E beyond its minimal requirement demonstrated a benefit on reducing age‐related visual decline. Additional clinical studies appear warranted.

### 4.10 Cognitive health

Canine cognitive dysfunction (CCD) is an analog of human dementia because the conditions may share similar neuropathologies such as cerebral atrophy, ventricular dilation, reduced vascularity, decreased levels of brain‐derived neurotrophic factor (BDNF) and accumulation of β‐amyloid (Aβ) plaques (Cotman et al., 2002; Dewey et al., 2019; Head et al., 2000). Clinically, CCD is characterized by an impairment in one or more cognitive domains such as learning and memory (the ability to perceive, store and retrieve information), executive function (the ability to plan and carry out cognitive tasks successfully), attention (the ability to concentrate on relevant stimuli) and visuospatial function (the ability to identify and integrate spatial and visual information), which may lead to behavioural changes. Confusion or disorientation, anxiety, disturbance of the sleep/wake cycle and decreased interaction with owners are clinical hallmarks of CCD (Dewey et al., 2019). Common complaints also include aggressive behaviours, inappropriate urination and defecation, and excessive vocalization, but these behaviours may not be directly caused by CCD. It was demonstrated that 74% of dogs over seven years old showed at least one potential clinical sign of CCD (Osella et al., 2007); however, CCD diagnostic criteria have not been established, and thus, the actual prevalence of CCD in senior dogs is currently unknown. Nonetheless, evidence is emerging demonstrating the ability of several plant‐based ingredients to benefit canine cognitive health.

Increased oxidative stress and inflammatory markers are detected in CCD brains as compared to healthy canine brains, a similar finding to that observed in humans (Head et al., 2002). Therefore, it has been hypothesized that nutrients with antioxidative and anti‐inflammatory properties play a role in the prevention of CCD. Twenty‐nine aged Beagles (>9 years old) were randomized into one of three diet groups: low antioxidant diet, moderate antioxidant diet (173 ppm vitamin E, <32 ppm vitamin C, 42 ppm L‐carnitine and <20 ppm lipoic acid) and high antioxidant diet (799 ppm vitamin E, 114 ppm vitamin C, 294 ppm L‐carnitine and 135 ppm lipoic acid) (Ikeda‐Douglas et al., 2004). All diets met AAFCO requirements. After 90 days of dietary intervention, dogs in both moderate and high antioxidant diet groups made significantly fewer errors on the landmark discrimination learning task than those in the control group, but only at levels with higher difficulty. In another trial, a higher proportion of healthy aged Beagles showed improvement in their working memory after receiving a mixed grape and blueberry extract supplement for 75 days as compared to those receiving no supplement (Fragua et al., 2017; Martineau et al., 2016).

A fortified diet containing spinach flakes, tomato pomace, grape pomace, carrot granules, citrus pulp and higher levels of lipoic acid, L‐carnitine, and vitamins C and E was used in three randomized controlled trials of healthy aged Beagles (>8 years old). In the first trial, 24 dogs were randomized to receive a control diet meeting the AAFCO standards or the fortified diet. After six months of dietary intervention, those fed the fortified diet learned the landmark discrimination learning task significantly more rapidly than those fed the control food (Milgram, Head, et al., 2002; Milgram, Zicker, et al., 2002). A higher proportion of dogs fed the fortified food also successfully completed the task when the difficulty level was increased. In the second trial, 23 dogs were assigned to receive either a control diet or the fortified diet (Milgram, Head, et al., 2002; Milgram, Zicker, et al., 2002). After six months of dietary intervention, the fortified diet group performed significantly better on the oddity discrimination learning task at higher, but not lower, levels of difficulty. Similarly, in another trial where 48 dogs were assigned to receive a control diet, the fortified diet, a behavioural enrichment intervention, or a combination of both the fortified diet and behavioural enrichment intervention (Milgram et al., 2004), those receiving a combined intervention performed significantly better on a size discrimination reversal‐learning task than the other three groups at the one‐year follow‐up. At the two‐year follow‐up, the combined intervention group also performed significantly better on the black/white discrimination learning task and the reversal‐learning task than the control diet group (Milgram et al., 2005). An analysis of brain samples from these Beagles suggested that the improvement of cognitive function, as a result of a combined dietary and behavioural interventions, was mediated by an increased expression of BDNF, a decrease of Aβ plaque deposits and an improvement of the antioxidant reserve system, resulting in a reduction of oxidative damage (Fahnestock et al., 2012; Head et al., 2000; Opii et al., 2008; Pop et al., 2010).

Nutrients hypothesized to be linked to cognitive decline in dogs include omega‐3 fatty acid DHA, vitamins B6 and B12, and folate (Pan et al., 2018). Older Beagle dogs (aged 9.1–11.5 years) were fed a supplemented diet consisting of DHA (0.58 g), EPA (0.50 g), arginine (6.05 g), selenium (0.13 mg), vitamins C (20.3 mg), E (132 mg) and B vitamins for six months (values reported per 1000 kcal) (Pan, Landsberg, et al., 2018); those receiving the supplemented diet performed significantly better on multiple phases of landmark discrimination learning tasks. While not all nutrients provided in the supplemented diet are necessarily of plant‐origin, vitamin C, vitamin E, arginine and B vitamins can be sourced from both animal and plant‐based ingredients. In another trial, a similar formula of the supplemented diet was given to aged dogs together with a medium chain triglyceride (MCT) solution at 6.5% or 9% for 90 days (Pan, Landsberg, et al., 2018). Dogs were evaluated for 6 signs of CCD, and significant improvements were seen in all CCD signs in dogs receiving 6.5% MCT diet (Pan, Landsberg, et al., 2018). MCT oil provides additional fats and ketone bodies, which may benefit brain function in dogs and is commonly sourced from plant oils, notably coconut and palm oils (Pan, Landsberg, et al., 2018; Pan, Landsberg, et al., 2018). However, it was noted that the 9% MCT diet was not well accepted by dogs in the study.

Results from these trials under a laboratory environment suggest that diets rich in antioxidative nutrients together with behavioural therapy may delay the onset of CCD in senior dogs as demonstrated by an improvement in learning, memory and visuospatial function after a long‐term dietary intervention (Ikeda‐Douglas et al., 2004; Milgram, Head, et al., 2002; Milgram, Zicker, et al., 2002; Milgram et al., 2004). However, it is unclear if the results are applicable to household dogs who are regularly exposed to a higher cognitively stimulating environment (Chapagain et al., 2018). Dietary and behavioural approaches aiming to prevent or delay the onset of CCD remain a focal recommendation since current medical treatments for CCD still result in a poor prognosis (Chapagain et al., 2018; Dewey et al., 2019).

## 5 DISCUSSION AND FUTURE DIRECTIONS

The trend of excluding plant‐based nutrients from canine diets prevents the inclusion of certain essential nutrients and phytonutrients, which have broad‐based effects on canine health. With theoretical examples of unbalanced diets (Table 2), we not only demonstrate how the inclusion of plant‐based foods, in proper amounts, does not compromise diet quality in terms of meeting the minimal requirements of essential amino acids and fatty acids, but we also highlight how the inclusion of plant‐based foods helps increase the content of some micronutrients (vitamins and minerals) that are generally low in some meat products. The profile of these essential and non‐essential nutrients is much more complex in foods than in individual component supplements. The term ‘non‐essential’ is somewhat misleading to the general public — although no intake requirements have been established for phytonutrients, it should not be interpreted that these compounds have no benefits on health. Rather than just meeting the minima that have been proven to be essential to maintain normal function, these phytonutrients may be useful for health optimization and longevity. Optimal nutrition represents a personalized strategy of disease prevention and health promotion. While providing optimal nutrition with a single dietary strategy is probably an unrealistic goal for the entire population because of the wide phenotypic and genotypic variation that exists, it is a more realistic aspirational goal for certain cohorts, such as companion dogs with identifiable risk factors for certain conditions. In this narrative review, we have accumulated current evidence, mainly from experimental and clinical trials, on the association between the consumption of plant foods or phytonutrients and various measures of canine health maintenance and promotion. Clearly, such work remains in its infancy and is tempered in some cases by experimental outcome measures, which may not be immediately clinically relevant. Nevertheless, documentation of biologic effects is often a first step in determining clinical relevance in the management of health and of disease.

Based on the current review of the literature, we have identified common limitations among existing studies and provided perspectives on addressing these issues. In order to advance the field of veterinary nutrition research as it relates to plant‐based ingredients, these important gaps need to be addressed:

● *Study sampling*. The number of animals included in the majority of the trials is relatively low and often statistically underpowered, especially when the effect of treatment on the measured outcomes is small. For example, in a replicated crossover trial with six dogs, polyphenol‐rich pomegranate peel extract had no apparent impact on nutrient digestibility (Jose et al., 2017). Similarly, no significant changes in body weight were observed in a parallel trial including six dogs per group, and the impact on other outcomes was inconclusive (Campigotto et al., 2020). Additionally, increased genetic heterogeneity, that is different breeds, among study subjects should be considered to enhance finding generalizability. For example, Beagles were the most heavily used breed among trials and the only breed included in all cognitive trials (Fragua et al., 2017; Ikeda‐Douglas et al., 2004; Milgram, Head, et al., 2002; Milgram, Zicker, et al., 2002; Milgram et al., 2004). Many studies were also performed under laboratory conditions, whereas household dogs are exposed to different environments (Chapagain et al., 2018). In addition to recruitment from veterinary clinics or hospitals (Hall, MacLeay, et al., 2016; Hall, Yerramilli, et al., 2016), we have previously demonstrated that a direct‐to‐consumer approach may be helpful for increasing both the number and the breed variety of household dogs in a research study (Jha et al., 2020).
● *Study end points*. Since many outcomes of interest are age‐related conditions, which may develop over a lifetime, there is a paucity of studies with clinically significant end points (such as disease rates, incidence, or severity). Various biochemical and molecular markers are frequently used as study end points, whose clinical interpretations are often ambiguous. Longitudinal studies, such as prospective cohort studies, where a group of participants are followed over time without any intervention, may be helpful to address this issue, as they are often used in humans to investigate the association between diet and risk of chronic diseases (Bao et al., 2016; Wong & Levy, 2013). Alternatively, case–control studies should be considered, especially with relatively rare conditions in the general population. An example of a retrospective case‐control study in 175 Scottish Terriers was previously discussed with respect to risk of TCC (Raghavan et al., 2005).
● *Nutritional intervention/exposure*. Unlike drugs, it is unlikely that a single nutrient at dietary doses could act as a panacea to a variety of chronic diseases (as compared to deficiency diseases where administration of a single nutrient may delay or reverse symptoms). Moreover, in order to determine the right dose of intake, a better understanding of nutrient bioavailability (which includes absorption, distribution, metabolism and excretion, often abbreviated as ADME) from either food sources or dietary supplements is warranted. If a study aims to investigate the biological functions of nutrients, there is a rationale to consider isolated nutrients as an intervention/exposure in the study. On the contrary, if the end point of the study is a complex clinical outcome, such as disease rate or physical performance, there is a higher clinical relevance to choose a more synergistic approach, such as a dietary shift, with or without other lifestyle modifications. Therefore, while more ADME studies are necessary to provide supporting evidence for proposed dietary shifts, combined supplements and dietary alterations are more clinically relevant as interventions on chronic disease outcomes.

While various plants have been well recognized as sources of nutrients (AAFCO, 2019; USDA ARS NDL, 2015), the use of plant‐based foods and dietary constituents for health benefits beyond meeting canine nutritional requirements is still a highly limited area of research. Phytonutrients possessing anti‐inflammatory and antioxidant properties may have notable roles in the prevention of chronic diseases, whose underlying development involves accumulated oxidative stress and chronic low‐grade inflammation or altered immune function (Khansari et al., 2009). Based on existing evidence, highlighted in this review, the potential of plant‐based ingredients and phytonutrients to benefit canine health is promising and warrants further investigation.

## CONFLICT OF INTEREST

JT, DET and JS are employees of and/or hold stock or stock options in NomNomNow Inc.

## ANIMAL WELFARE STATEMENT

The authors confirm that the ethical policies of the journal, as noted on the journal's author guidelines page, have been adhered to. No ethical approval was required as this is a review article with no original research data.
